# Treatment outcomes and their trend among tuberculosis patients treated at peripheral health settings of Northern Ethiopia between 2009 and 2014: a registry-based retrospective analysis

**DOI:** 10.1186/s13104-019-4824-9

**Published:** 2019-12-02

**Authors:** Mahmud Abdulkader, Ischa van Aken, Selam Niguse, Haftamu Hailekiros, Mark Spigt

**Affiliations:** 1 Department of Medical Microbiology and Immunology, School of Medicine, Mekelle University, Mekelle, Ethiopia; 2 Department of Family Medicine, Maastricht University, Maastricht, The Netherlands

**Keywords:** Treatment outcome, Tuberculosis, Trend analysis, Ethiopia

## Abstract

**Objective:**

Evidence on treatment outcomes and their trend analysis through a register based retrospective study have significant contributions in the improvement of a national tuberculosis program. This study was aimed at determining tuberculosis treatment outcomes and their trend analysis.

**Results:**

A total of 3445 patient records were included. More than half (58%) were males and the mean age was 33.88 ± 16.91 years (range 0–90). From the total TB patients, 18.8% were HIV co infected. The treatment outcome of TB patients were 371 (10.8%) cured, 2234 (64.8) treatment completed, 119 (3.5%) died, 9 (0.3%) failed, 178 (5.1%) defaulted and 534 (15.5%) were transferred out. The overall treatment success rate was 89.5%. When assessed on yearly basis, treatment success rate was 87% in year 2009–2010 to 92.8% in 2013–2014 with 6.67% change in the outcome indicator over the 5 years period. Among pulmonary TB, pulmonary negative TB and extra pulmonary TB, the rate of successful treatment outcome was 83.1% to 89%, 85.1% to 89.4%, and 87.4% to 92%, respectively in the year 2009–2010 to 2013–2014. The percentage of the overall successful treatment outcomes were significantly associated with the year of treatment (p = 0.014).

## Introduction

Tuberculosis (TB) is placed as one of the top ten and third cause of morbidity from all diseases and infectious disease, respectively. Every day, around 5000 TB related deaths are recorded, of which, 95% occur in low- and middle-income countries (LMICs) [1]. Moreover, multidrug/rifampicin-resistant TB (MDR/RR-TB) is becoming a public health crisis and health security threat [2]. The main strategy to control TB relies on early diagnosis and prompt treatment initiations [3]. WHO has implemented a standardized directly observed treatment, short-course (DOTS)/Stop TB Strategy to scale up TB prevention and control. Despite the efforts made under the global End TB strategy, a significant number of people are unknown to the health system or are not receiving proper treatment. Majority of these belongs to peripheral health settings of LMICs [4, 5]. For example TB treatment saved about 54 million lives globally between 2000 and 2017, but important diagnostic and treatment gaps persist. TB treatment success rate for people with TB was 83% in 2016. Most of the gaps in detection and treatment were in WHO African region, where the burden of HIV associated TB is highest [6]. Studies have indicated that access to treatment, poor socioeconomic status, health service access and use, treatment seeking behavior and poor knowledge about the disease affects treatment outcomes [7].

Ethiopia is among the 22 high TB and 30 high MDR-TB burden countries [1]. In Ethiopia, the incidence of TB and MDR/RR-TB in 2018 was 164 and 5.2 per 100,000 populations, respectively [8]. The TB control program in Ethiopia introduced health facility-based DOTS as a pilot program in 1992. Since then, a number of patients were enrolled to the national TB program. Though Ethiopia national TB program is working under the global strategy, there are challenges in the success of the national TB program [9]. WHO recommends that treatment outcome analysis should be carried out every year at national and district levels [10]. Hence, determining treatment outcome and performing trend analysis will in turn contribute to the improvement of TB control programs and to the decrease of TB morbidity and mortality. In Ethiopia considerable number of studies has been conducted, showing different TB treatment outcomes [11–19]. However, further information is required on the outcomes of TB treatment in all forms of TB and all age groups and on the trend of the outcome. Therefore, this study was aimed at assessing tuberculosis treatment outcomes and determining their trend in all forms of TB patients that have been treated for TB in the health settings.

## Main text

### Methods

#### Study setting

The study was conducted in three peripheral health facilities; Mekelle, Semen and Kasech Health Centers of Mekelle, in Northern Ethiopia. The study area is located 783 kms to the North of the capital city of Ethiopia, Addis Ababa. The total population of Mekelle has been estimated to be around 216,000 in 2007 [20]. Selected health facilities were the biggest facilities in terms of TB care and treatment provision with highest patient flow and were located in the relatively high performer zone with regard to TB case detection [21]. Based on the TB clinics’ registration log books, on average 230, 300 and 160 patients per year were estimated to be receiving TB treatments in Mekelle, Semen and Kassech Health Centers, respectively.

#### Study design and data collection procedure

The study design was a register based retrospective case series analyses on patients of all TB forms who received TB therapy between July 2009 and July 2014. A structured data extraction form was prepared and used to collect information on socio-demographic characteristics (such as age and sex) and clinical-related data (such as, TB history, TB type, date starting treatment, HIV status, date end of treatment and treatment outcome). The data source was the TB registers at the TB clinics of the health facilities, where all patients diagnosed for active TB disease are put on anti-TB treatment and monitored throughout the treatment course. The inclusion criteria were having a complete and readable record in the TB registration book and patients that were diagnosed as non TB after starting of treatment were excluded from the study.

#### Operational definition

Treatment outcome was defined according to the existing WHO definitions at the time of data collection [22]. Accordingly, components were defined as follows: *cured*- pulmonary TB patient with smear or culture negative in the last month of treatment and at least one previous occasion. Treatment completed—A TB patient who completed treatment without evidence of failure but with no record to show that sputum smear or culture results in the last month of treatment and on at least one previous occasion were negative, either because tests were not done or because results are unavailable. *Treatment failed*—is a TB patient whose sputum smear or culture is positive at month 5 or later during treatment. *Died*—is defined as TB patient who dies for any reason before starting or during the course of treatment. *Lost to follow up*—A TB patient who did not start treatment or whose treatment was interrupted for 2 consecutive months or more. *Not evaluated*—A TB patient for whom no treatment outcome is assigned. This includes cases “transferred out” to another treatment unit as well as cases for whom the treatment outcome is unknown to the reporting unit. *Treatment success*—was defined as the sum of cured and treatment completed. In this study the findings of transferred out patients were not included as one of the TB treatment outcomes.

#### Statistical analysis

SPSS version 22 for Windows was used for data analysis. Descriptive statistics were used to summarize patients, characteristics across the outcomes variables. Trend analysis was performed using the percentages of successful treatment outcomes. Moreover, percentage changes in outcome indicators for 5 years period was analysed. Trend p-value less than 0.05 were considered as statistically significant.

### Results

**Fig. 1:**
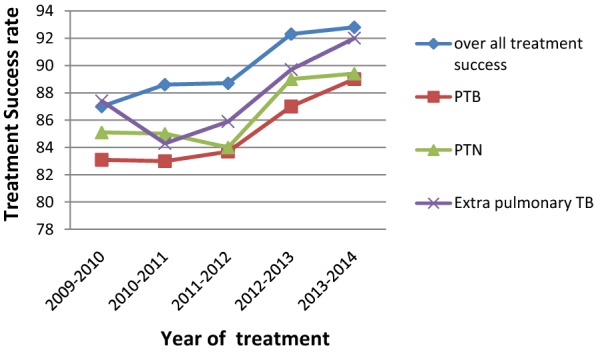
Trend analysis of treatment success rate. *PTB* pulmonary tuberculosis, *PTN* pulmonary tuberculosis negative

A total of 3445 patient cards were included from the three health centers, of which 810 (23.5%) were from Kassech, 1143 (33.2%) from Mekelle and 1492 (43.3%) from Semen health centers. Majority, 1999 (58%), were male. The mean age and standard deviation of the study population was 33 ± 16.91 years (range 0–90 years) and most, 2833 (82.3%), were adults. Patients with extra-pulmonary TB constitute the largest proportion 1471 (42.7%), followed by pulmonary negative 1357 (39.4%) and pulmonary positive 617 (17.9%) patients. Majority of the patients 2602 (91.8%) were new patients. Out of the total TB patients analysed only 1403 (40.7%) had a known HIV status result, among which, 652 (18.8%) of the TB patients were co-infected with HIV (Table 1). Among the study population, 371 (10.8%) were cured, 2234 (64.8%) had completed their treatment, 119 (3.5%) died, 9 (0.3) failed, 178 (0.1%) defaulted and 534 (15.4) were transferred out. In pulmonary positive patients, 364 (71.9%) were cured, 70 (13.8%) had completed their treatment, 20 (4.0%) died, 8 (1.5%) failed and 44 (8.7%) were defaulters. The overall number of patients with treatment success was 2605 (89.5%). Of the different TB types, pulmonary positive patients had a treatment success rate of 434 (85.6%), followed by pulmonary negative 1033 (89.4%) and extra-pulmonary 1136 (90.7%) patients (Table 2). The treatment success rates for newly treated TB patients and TB patients co-infected with HIV were 89.5% and 85.2%. The trend of overall treatment success rates in the year 2009–2010, 2010–2011, 2011–2012, 2012–2013 and 2013–2014 were 87%, 88.6%, 88.7%, 92.3%, and 92.8%, respectively. The overall treatment success rate was 87% in year 2009–2010 to 92.8% in 2013–2014 with 6.67% change in the outcome indicator over the 5 years period. Among pulmonary TB, pulmonary negative TB and extra pulmonary TB patients the trend of successful treatment rate was observed to increase from 83.1% to 89%, 85.1% to 89.4%, and 87.4% to 92%, respectively, in the year 2009–2010 to 2013–2014 (Fig. 1). The percentage of overall successful treatment outcomes were significantly associated with the year of treatment (p = 0.014). However, there was no significant association in the trend of successful treatment outcomes among patients with pulmonary TB (p = 0.05), pulmonary negative TB (p = 0.32) and extra pulmonary TB (p = 0.14).

**Table 1:** Demographic and clinical characteristics of study population

Variables	N (%)
Health center	
Kassech	810 (23.5)
Mekelle	1143 (33.2)
Semen	1492 (43.3)
Age group	
0–17	350 (10.2)
18–64	2833 (82.2)
65 +	262 (7.6)
Sex	
Male	1999 (58)
Female	1463 (42)
TB type	
Pulmonary positive	617 (17.9)
Pulmonary negative	1357 (39.4)
Extra-pulmonary	1471 (42.7)
TB history category^a^	
New	2602 (91.8)
After relapse	122 (4.3)
After failure	12 (0.4)
After default	3 (0.1)
Other	94 (3.3)
HIV status^b^	
Negative	751 (75.0)
Positive	652 (18.8)

^a^ Excluding Transfer-in

^b^ Excluding patients with Unknown status and missed values [2042 (59.3%)]

**Table 2:** Treatment outcome among TB patients in the peripheral settings of Ethiopia

Variables	Overall treatment outcome^a^ (*n* = 2911) n (%)	TB type^a^		
		Pulmonary positive (*n* = 506) n (%)	Pulmonary negative (*n* = 1155) n (%)	Extra-pulmonary (*n* = 1250) n (%)
Cured	371 (12.7)	364 (71.9)	5 (0.4)	1 (0.0)
Treatment complete	2234 (76.7)	70 (13.8)	1028 (89)	1135 (90.8)
Died	119 (4.1)	20 (4.0)	47 (4.1)	55 (4.4)
Failed	9 (0.3)	8 (1.6)	1 (0.0)	0 (0.0)
Default	178 (6.1)	44 (8.7)	74 (6.4)	59 (4.7)

^a^ Excluding 534 (15.4%) Transfer-out

### Discussion

Over all, an increase in treatment success rate was observed over the studied years and a relatively lower success was recorded for HIV co-infected TB patients. Similar studies conducted on TB/HIV co-infected patients have reported lower successful treatment outcome among these patients [23–25]. The overall treatment success rate was 89.5% and increased from 87.0% to 92.8% between the years analysed (2009–2010 and 2013–2014). This increase was in line with the Annual Performance Report of the Ministry of Health which was shown to increase from 84% in 2010 to 92% in 2014 [26]. The possible reason might be due to improved adherence of patients to anti-tuberculosis treatment. The overall successful treatment outcome recorded in the present study was in agreement with previous studies conducted in Ethiopia and elsewhere [13, 18, 27–30]. Our result was higher than the national pooled estimates (68.1%) calculated for six European countries [31]. In this report, a cure rate of 69.1% was found in pulmonary positive patients. Difference in treatment outcomes could be due to variations in DOTS performance. Other reasons could be due to differences in duration of study period, sample size and in the study settings.

The proportion of HIV/TB co-infection (18.8%) in the present study was higher than the national average (10.7%) [26], but was lower than (29.4% and 34.7%) other reports from the Ethiopia [32] and from Cameroon (35.6%) [33]. In the Sentinel Surveillance Report, HIV/TB co-infection rate of 17.2% was reported from Tigray region in 2013/2014, with the highest rate (34.7%) to be reported from Addis Ababa [26]. New TB treatment cases had higher rates of successful treatment outcome. Our finding was in agreement with other studies conducted in Somalia [30], Nigeria [34] and other countries [35, 36]. Furthermore, being HIV positive resulted into lower treatment success rates. This finding supports other similar findings suggesting that HIV co-infected TB patients have significantly lower cure rates and lower treatment success rates compared to non-HIV infected counterparts [25, 27, 37]. Viral co-infection might increase the risk of anti-tuberculosis treatment-induced hepatotoxicity leading to frequent discontinuation of the first-line anti-tuberculosis drugs and hence lower treatment adherence and lower cure rates [38].

In conclusion, findings from this study show good treatment outcome of patients which is in line with Ethiopian national TB program report. Early detection of TB with prompt initiation of effective treatment of anti-TB drugs alongside the scaling up of HIV prevention activities are crucial to increase successful treatment outcome among TB patients.

## Limitations

One of the major limitations of this study was the use of retrospective secondary data, which is limited to whatever documented in the TB registers of the TB clinic. Variables such as treatment adherence and other disease conditions which might affect outcome were not captured in the patient’s file. Furthermore, selected sites encompass small geographic region and hence patients from these facilities might have a different profile from patients residing in other parts of the country. Another limitation might emerge from the study design where we cannot make causal inferences regarding the efficacy of the investigated treatment and the internal validity might be low due to the lack of comparator group.

## Data Availability

The datasets used and/or analysed during the current study are available from the corresponding author on reasonable request.

## Abbreviations

ART: antiretroviral therapy
DOTS: directly observed treatment-short course
HIV: human immunodeficiency virus
LTBI: latent tuberculosis infection
TB: tuberculosis
WHO: World Health Organization
